# Cloning and expression of *Toxoplasma gondii* GRA-4 recombinant protein as a toxoplasmosis diagnostic kit candidate

**DOI:** 10.14202/vetworld.2020.2085-2091

**Published:** 2020-10-05

**Authors:** Muhammad Hanafiah, Teuku Zahrial Helmi, Amalia Sutriana, Dwi Priyowidodo, Fihiruddin Fihiruddin

**Affiliations:** 1 Parasitology Laboratory, Faculty of Veterinary Medicine, Universitas Syiah Kuala, Banda Aceh, Indonesia; 2 Laboratory of Biochemistry, Faculty of Veterinary Medicine, Universitas Syiah Kuala, Banda Aceh, Indonesia; 3 Pharmacology Laboratory, Faculty of Veterinary Medicine, Universitas Syiah Kuala Banda Aceh, Indonesia; 4 Department of Parasitology, Faculty of Veterinary Medicine, Gadjah Mada University, Yogyakarta, Indonesia; 5 Department of Medical Laboratory Technology, Politeknik Kemenkes Mataram, Sandubaya Mataram Nusa Tenggara Barat Indonesia

**Keywords:** cloning, expression, GRA-4, pET-SUMO, plasmid, recombinant

## Abstract

**Aim:**

The objective of this study was to produce recombinant protein GRA-4 (rGRA-4) of a local *Toxoplasma gondii* isolate as a candidate for a toxoplasmosis diagnosis kit in *Escherichia coli* BL21 (DE3) competent cells using pET SUMO plasmid.

**Materials and Methods:**

Samples used were stock *T. gondii* tachyzoites DNA from the Parasitology Laboratory, Faculty of Veterinary Medicine, Gadjah Mada University, Yogyakarta. Amplified GRA-4 polymerase chain reaction product of *T. gondii* tachyzoite DNA was cloned in the pET-SUMO TA^R^ cloning vector. The *GRA-4* gene from *T. gondii* local isolate was sequenced, followed by plasmid transformation, recombinant plasmid DNA isolation, and recombinant protein expression in DE3 competent cells.

**Results:**

The amplification product of *GRA-4* *T. gondii* gene was 1036 bp, with 48 kDa molecular weight after expression in DE3 competent cells. An alignment of the amino acid sequence of GRA-4 from the local isolate which was cloned with GRA-4 was obtained from NCBI database and showed 99.61% homology to the predicted GRA-4 from the *T. gondii* Izatnagar isolate. Amino acid sequence of the predicted GRA-4 protein from local isolate was different at positions 19 and 304.

**Conclusion:**

This research cloned rGRA-4 in pET SUMO plasmid.

## Introduction

*Toxoplasma gondii* is a protozoan parasite that infects all mammals and birds and causes toxoplasmosis. The prevalence rate of *T. gondii* infection varies between countries, with infection rates between 10% and 80% of the population [1]. Moreover, animals consumed by humans are often infected with *T. gondii* muscle cysts, a source of parasite transmission to humans, which remain a public health concern [2]. Toxoplasmosis diagnosis consists of either a serological test using monoclonal antibody (fluorescent antibody, ELISA), polymerase chain reaction (PCR), molecular probes (DNA tracking), or dipstick test, in conjunction with clinical symptoms. *Toxoplasma* antigens have been detected in several domestic cat organs using immunohistochemistry with antigen labeled-(strept) Avidin-Biotin (LAB-SA) [3]. Hanafiah *et al*. [4] also detected 33.3% toxoplasmosis prevalence in cats using the PCR technique. However, the use of recombinant GRA-4 (rGRA-4) protein as a potential antigen in a diagnostic kit has never been tested.

*Toxoplasma* diagnosis often uses antigens from research animals infected with *T. gondii*, but the method was recently banned due to animal welfare concerns [5]. As a solution, DNA recombinant technology can provide large amounts of immunogenic protein, allowing promising molecular based diagnostics and vaccine developments, and increasing feasibility and sustainability. The dense granule protein (GRA) is the main component of *T. gondii* vacuoles, which protect tachyzoites, the cyst wall, and bradyzoites, against the host humoral and cellular immune responses [6]. Recombinant antigen protein TgPI-1 (rTgPI-1), ROP2 (rROP2) and GRA-4 (rGRA-4) can easily be expressed and purified [7-9].

Here, the rROP2 (residues 96-561) and rGRA-4 proteins were expressed in *Escherichia coli* strain M15 (QIAGEN) and purified under non-denaturing condition on Ni2+-NTA agarose column, (QIAGEN), as described previously [7,10]. The objective of this research was to produce *T. gondii* recombinant antigen GRA-4 in *E. coli* BL21 (DE3) competent cells as a candidate for a toxoplasmosis vaccine and diagnostic kits.

## Materials and Methods

### Ethical approval

This study was approved by Veterinary Ethics Committee Faculty of Veterinary Medicine Universitas Syiah Kuala, Banda Aceh, Indonesia (Approval No. 43/KEPH/IXVH/2019).

### Study location and period

The research was performed in the Parasitology Laboratory, Faculty of Veterinary Medicine, Universitas Syiah Kuala and Inter-University Research Center, Gadjah Mada University (UGM), Yogyakarta, Indonesia. This study was conducted in April 2019.

### Amplification of *T. gondii* tachyzoite DNA by PCR

DNA from *T. gondii* tachyzoites was obtained from the Parasitology Laboratory, Veterinary Medicine Faculty, UGM, Yogyakarta. The forward primer and reverse primers were designed according to 1036 bp sequence obtained from GenBank (https://www.ncbi.nlm.nih.gov/genbank/), accession number M76432.1, utilizing the Gene Runner Software. Specific primers for the gene encoding the GRA-4 protein were forward primer GRA-4: 5’-ATG CAG GGC ACT TGG TTT TC-3’ and reverse primer GRA-4: 5’-TCA CTC TTT GCG CAT TCT TT-3’. The primers were diluted to 10 pmol/μL concentration. The mixture for the amplification process was made by adding components into pure the Taq RTG-PCR as follows: 2 μL template DNA, 2 μL GRA-4 forward primer, and 2 μL GRA-4 reverse primer, 12.5 μL *Taq* polymerase (Biotaq^®^, Bioline, London, UK), and 6.5 μL nuclease free water, to make a total volume of 25 μL.

Amplification utilized a thermocycler with the following settings: Denaturation at 94°C for 5 min; annealing at 52°C for 40 s, and extension at 72°C for 2 min; followed by a final extension at 72°C for 5 min, and cooling at 25°C. All reactions were performed for 30 cycles. The amplification products were then processed for electrophoresis in 1% agarose gel at 100 volts for 35 min. Electrophoresis results were read using a transilluminator viewer.

### Nucleotide sequence of the *T. gondii* GRA-4 gene local isolate

The resulting PCR product of the *T. gondii* *GRA-4* gene was sequenced to compare the nucleotide sequences of the obtained gene with the *T. gondii* *GRA-4* gene deposited in GenBank. Sequencing was also performed on the *GRA-4* gene following insertion into pET SUMO plasmid sequence to confirm the correct position and frame. The Champion™ pET SUMO Expression System was provided as a complete system. The Champion™ pET SUMO TA Cloning^®^ box contains linearized Champion™ pET SUMO vector, sterile water, dNTPs, 10× PCR Buffer, control template and primers, T4 DNA ligase, 10× ligation buffer, primers for sequencing or PCR screening, and an expression control and stored at −20°C. The SUMO Protease box contains SUMO Protease and buffers and stored at −80°C. The One Shot^®^ TOP10 box contains 21 50 μL aliquots of chemically competent *E. coli*, SOC medium, and a control plasmid and stored at −80°C. The One Shot^®^ BL21(DE3) box contains 21 50 μL aliquots of chemically competent *E. coli*, SOC medium, and a control plasmid and stored at −80°C. Sequencing was performed in the Integrated Research and Testing Laboratory, UGM, Yogyakarta.

### Cloning of the *T. gondii* GRA-4 gene in the pET-SUMO plasmid

Fresh PCR product with a length of 1036 bp was ligated to the pET-SUMO vector in the following ligation reaction: 2 μL PCR product, 1 μL ligation buffer, 2 μL plasmid pET SUMO, 4 μL nuclease free water, and 1 μL of T4 DNA ligase (Invitrogen, Thermo Fischer, USA), in a total volume of 10 μL. The mixture was transferred into a microtube and incubated at 15°C for 4 h or overnight [11].

### Plasmid transformation

To transform bacteria, 10 μL of the cloning reaction was mixed with competent *E. coli* cells (One Short^®^ Mach1™-T1^R^ Invitrogen, Thermo Fischer, USA) and incubated for 30 min on ice. Competent cells were heat-shocked for 30 s at 42°C, and quickly placed on ice for 1-2 min, followed by the addition of 250 μL SOC medium. The tube was shaken at 200 rpm at 37°C for 1 h. The mixture was then plated on Luria Bertani (LB) agar, which contained 50 μg/mL kanamycin and incubated at 37°C for 24-48 h.

### Recombinant plasmid DNA isolation

Isolation of DNA plasmid was performed according the protocol provided in the Presto^™^ Mini Plasmid kit (Geneaid, New Taipei City, Taiwan).

### Amplification of *T. gondii* GRA-4 gene inserted from pET SUMO plasmid

The amplification of *T. gondii GRA-4* gene from the pET SUMO plasmid was performed using the GRA-4 forward and GRA-4 reverse specific primers and the plasmid SUMO forward and T7 reverse primers. *T. gondii* recombinant GRA-4 DNA was amplified by preparing a master mix solution of 25 μL for each sample including 12.5 μL of PCR master mix reaction solution, 2 μL each of the GRA-4 forward and reverse primer, and along with 2 μL of the SUMO forward primer and 2 μL of the T7 reverse primer, at a concentration of 10 pmol/μL for each primer. The mixture included 6.5 μL of dH_2_0 and 2 μL plasmid template.

The amplification process used a thermocycler with the following conditions: Initial denaturation at 94°C for 5 min, denaturation at 94°C for 1 min, annealing at 72°C for 1 min, extension at 72°C for 1 min, and final extension at 72°C for 5 min, for 30 cycles.

### Recombinant protein expression

Purified plasmid containing the *T. gondii* *GRA-4* gene was then transferred into DE3 competent cells as described previously. The transformed bacteria were then cultured in 10.0 mL of liquid LB and incubated at 37°C for 24 h. A total of 10% bacteria culture was transferred into liquid LB media and incubated for 4 h in a shaker (180 rpm) until the culture reached 0.6 optical density. Into the culture, 1 mM isopropyl β-d-1-thiogalactopyranoside was added and incubated for 2 h. Induced cultured bacteria were transferred into two conical tubes (50 mL each) and centrifuged (4°C) at 5000 rpm for 10 min. The supernatant was discarded, and the pellet was resuspended, followed by dilution to 50 mL. The diluted bacteria were then centrifuged (4°C) at 5000 rpm for 10 min. The washing process was repeated twice. The supernatant was discarded and PBS I pH 7.0 was added into the pellet. The tube was sonicated 6 times for 30 s. After sonication, a protein sample was added to a 1.5 mL tube. The microtube was centrifuged in a 4°C centrifuge at 10,000 rpm for 5 min. The resulting supernatant was transferred into a new tube, the protein was stored in −20°C and the pellet was discarded.

### Purification and examination of protein using SDS-PAGE

Preparation of 15% gradient gel and 3% stacking gel was carried out for protein electrophoresis purpose. Gradient gel was allowed to flow to slab gel to a certain level and added with butanol to cover the surface of gradient gel solution. Polymerization of gradient gel occurs for 4-5 h, and then the gel surface was rinsed with aquadest to remove the remaining butanol. Gradient gel was overlaid with stacking gel, and the comb was inserted and left for 30-40 min. The loading dye was loaded to a protein sample with a ratio of 1:4 (10 μL loading dye: 40 μL sample) in a tube, homogenized, and heat the sample in boiling water at temperature of −80°C for 5 min. After heating, the sample was cooled, and then loaded in the gel wells of electrophoresis apparatus. The electrophoresis was run at 120 V for 2 h before stained with coomassie blue for 1 h using a shaker. The gel was rinsed with ddH2O, and then further washed using de-staining solution for 30 min until the clear bands were formed. Finally, the gel was rinsed with aquadest to remove the remaining color. Purification process was conducted using Nickel Affinity Gel Column Chromatography due to Ag85 is fusion protein consisting polyhistidine at N-terminal end. Procedure of purificaion was performed using protocol in Protino™ Ni-TED 2000 which has been modified in elution volume.

## Results

### Amplification of *T. gondii* tachyzoite DNA by PCR

**Figure-1:**
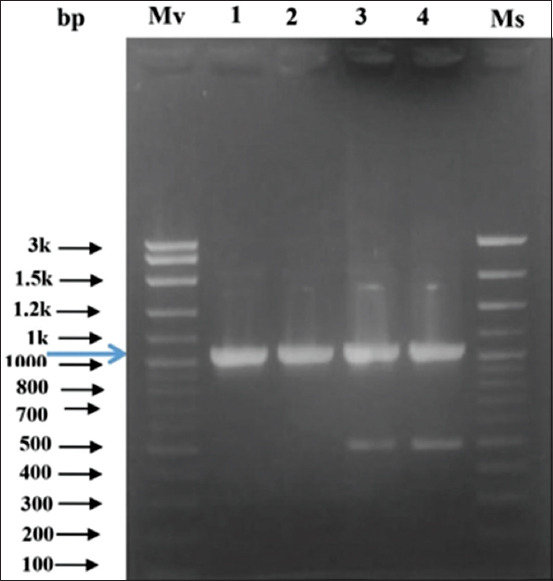
DNA amplification results from *Toxoplasma* *GRA-4* gene in 1% agarose gel. Mv=Marker vivantis (1 kb DNA ladder); lane 1= DNA in 5× dilution (Mix Go Tag); lane 2=DNA in 10× dilution (Mix Go Tag); lane 3=DNA in 5× dilution (Bio line); lane 4=DNA in 10× dilution (Bio line); Ms=Marker smobio (1 kb DNA ladder).

Genomic DNA of the local isolate of *T. gondii* tachyzoites was amplified by PCR and analyzed by agarose gel electrophoresis. The PCR product showed one band with a molecular weight of approximately 1038 bp (Figure-1).

### Nucleotide plasmid sequence and DNA recombinant

Sequence analysis of the isolated *GRA-*4 gene was described previously [12] and sequence of the plasmid confirmed that there were no amplification errors in the cloned *GRA-4* gene sequence. The sequence was also confirmed by comparing the database entry using the Basic Local Alignment Search Tool (BLAST) software.

After conducting BLAST runs for the predicted amino acid sequence using sequence deposited in GenBank, the sequence of GRA-4 gene from the local isolate showed homology with several *T. gondii* GRA-4 genes as presented in Table-1.

**Table 1:** Homology of *GRA-4* gene samples isolate with others *Toxoplasma gondii* in NCBI database.

No.	Bacteria species	Query (%)	Coverage E. value	Identity (%)	Accession
1.	*T. gondii* dense granule (*GRA-4*) gene, complete cds	100	0.00	100	M76432.1
2.	*T. gondii* isolate Izatnagar *GRA-4*-like gene	99	0.00	99.61	EU660037.1
3.	TPA: *T. gondii* VEG, chromosome chrXI complete genome	100	0.00	97.69	LN714501.1
4.	*T. gondii* ME49 dense granule protein 4 (GRA-4) mRNA	100	0.00	97.69	XM 00236 4283.1
5.	*T. gondii* cDNA clone: XTG04280.2 full cDNA	85	0.00	98.08	AK317865.1

T. gondii=Toxoplasma gondii

### GRA-4 protein clone in pET-SUMO plasmid

**Figure-2:**
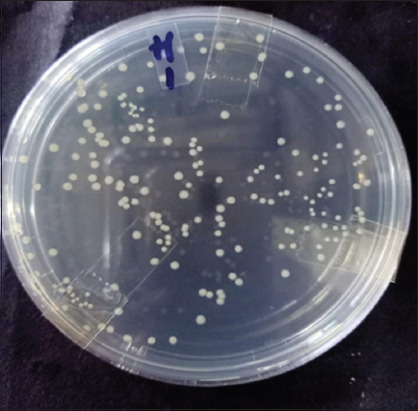
Bacterial colony of MUC I.

The MUC I *E. coli* was successfully transformed with the GRA-4-containing pET SUMO plasmid and appeared as colonies on agar plates, as shown in Figure-2.

### Plasmid transformation

**Figure-3:**
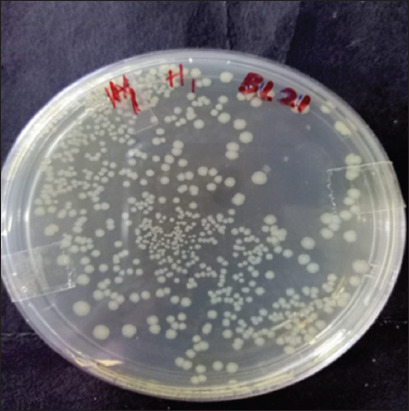
The culture of BL 21. The colony was assumed as recombinant *Escherichia coli* BL21 bacteria.

Cultures of *E. coli* BL 21 transformed bacteria are shown in Figure-3.

### Recombinant plasmid DNA amplification

**Figure-4:**
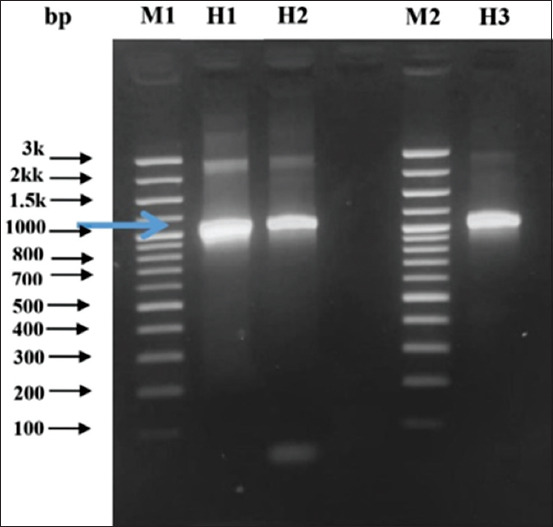
Recombinant plasmid DNA 1. DNA amplification results from *GRA-4* gene of *Toxoplasma* in 1% agarose gel. M1=Marker 100 bp, H1=DNA in 5× dilution (Mix Go Tag); H2=DNA in 10× dilution (Mix Go Tag); M2=Marker 100 bp; H3=DNA Tachyzoite *Toxoplasma* (control). Arrow showed the recombinant plasmid DNA molecular weight.

Agarose gel electrophoresis of PCR products obtained from the recombinant plasmid DNA. Figure-4 shows that the *GRA-*4 gene was a result of cloning inserted into pET SUMO vector. The used of pET SUMO vector as a cloning vector is controlled by bacteriophage T7 promotor. The target gene Ligation that is PCR product in pet sumo is conducted without using restrictions enzymes, since pET SUMO has 3’ *deoxythymidine* (T) single residue which able to be directly inserted by PCR product into the *multiple cloning site* located between sumo and T7 terminator [13].

### Recombinant protein isolation

**Figure-5:**
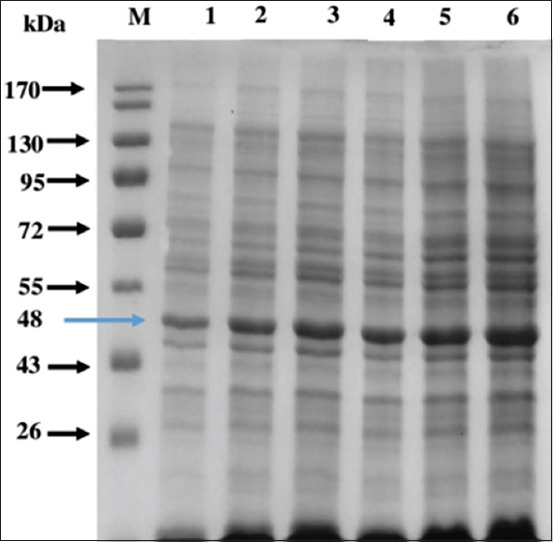
SDS PAGE electrophoresis of recombinant GRA-4 *Toxoplasma gondii* protein. M=Marker (Sigma); line 1=Supernatant protein (control); line 2=Supernatant H1; line 3=Supernatant H2; line 4=Pellet protein (control); line 5=Pelet H1; line 6=Pelet H2. Each 20 μL. Arrow showed the recombinant protein molecular weight.

The recombinant protein isolation product was then examined by SDS-Page. The results displayed the protein band with a size of 48 kDa both in supernatant and protein pellet. Figure-5 shows the SDS page results of the recombinant GRA-4 protein .

## Discussion

To produce *T. gondii* recombinant antigen, DNA from a tachyzoite sample was amplified using specific primers for GRA-4, which produced a 1038 bp product. The resulting DNA product was identical in size to the DNA amplified from the recombinant GRA-4 plasmid obtained from *E. coli*. These results show that *GRA-4* gene was successfully cloned into the pET SUMO plasmid (Figure-1).

Gene cloning can routinely be performed using the PCR method, which allows a clone to carry a specific desired gene [14-16]. This study cloned the *GRA-4* gene into the pET-SUMO (Figure-2) plasmid. Successful cloning using a PCR product is influenced by several factors: The purity of PCR product, the choice of restriction endonuclease enzyme, the creation of primer, and the choice of plasmid which is used as a PCR product carrier vector [17]. Although it has a low success rate, this cloning method has often been employed by adding restriction endonuclease enzyme into the primer mix, and using a relatively small plasmid vector (2.9-5 kb).

The *T. gondii GRA-4* gene has been cloned previously by Marjanh *et al*. [18], who successfully inserted GRA-4 into the pPICZα A expression vector alongside *Picnic*. The *GRA-4* gene was also previously cloned into the pcDNA3 expression vector, which can express protein in CHO eukaryote cells. Using this GRA-4 expression system, a recombinant vaccine has been produced and the ability of pGRA-4 to induce a protective immune response has been evaluated in model rats.

To produce a recombinant vaccine, *T. gondii* *GRA-4* gene expression must be confirmed in eukaryotic cells (CHO). Successful *GRA-4* gene cloning in the pcDNA3 expression plasmid was confirmed using a restriction enzyme and PCR. *GRA-4* gene expression was further examined *in vitro*. Recombinant pcGRA-4 plasmid was transfected into CHO eukaryote cells using the calcium phosphate method and analyzed by SDS-PAGE. The molecular weight of the resulting protein was 42 kDa on a Western blot. However, when the pET-32a expression vector was used for *GRA-4* gene expression in a prokaryotic system, the reported molecular weight was approximately 50 kDa [18]. Furthermore, the protein produced in this study was antigenic as it was recognized by human antibody positive serum from a patient with an acute toxoplasmosis infection.

Indrasanti *et al*. [19] reported the efficacy of rGRA-4 and rROP2 in inducing immunity in combination with alum. This data suggest that a multi-antigen *T. gondii* vaccine may be possible by utilizing alum as a supporting compound. This study also showed that the *T. gondii* *MIC3* gene clone can successfully been cloned in *E. coli*.

Here, recombinant DNA transformation into DE3 bacteria (Figure-3) was performed using the heat shock technique, which transfers ligation product recombinant DNA into a competent host, DE3 [15]. Competent bacteria were obtained using cold CaCl_2_ treatment. Transformation was effectively performed at 42°C for 90 s [16,20]. The transformation mechanism involving CaCl_2_ and heat was assumed to damage the bacterial cell wall, which eased the access of the plasmid into the bacteria [20].

The transformed cells were then cultured on an LB agar plate, which contained ampicillin as a selectable marker. The number of potential recombinant colonies was numerous; however, confirmation by Western blot is required. At the cellular level, gene expression of foreign DNA may have detrimental effects on the host, even if the translation product of the respective gene is not toxic. Foreign gene expression may be detrimental to recombinant bacteria in the form of reduction of cell growth and lowering cell stability compared to non-recombinant cells and may also cause morphological changes such as an increase in cell fragility [21,22].

Recombinant plasmid analysis was conducted using the PCR method and the specific primers GRA-4 F and GRA-4 R, followed by agarose gel electrophoresis on a 1% agarose gel (Figure-4). The amplification result was positive for recombinant plasmid, which was shown by a similar band as a positive control.

An alignment of the amino acid sequence of the GRA-4 from the local isolate, which was cloned here, and the predicted GRA-4 obtained from GenBank showed 99.61% homology to predicted amino acid for the *GRA-4* gene of the *T. gondii* Izatnagar isolate. The amino acid sequence predicted from the *GRA-4* gene from the local isolate was different at positions 19 and 304. As compared to the predicted GRA-4 from all isolates observed in the NCBI databases, the predicted protein sequence of the GRA-4 from the local isolate coded for a different amino acid at position 326 (Asp to Ser substitution). Amino acid differences between the *T. gondii* *GRA-4* gene from local isolate and the *T. gondii* VEG, *T. gondi* ME49, and *T. gondii* cDNA clone XTG04280.2 were found at positions 28, 36, 39, 43, 45, 58, 65, 69, 98, 117, 154, 192, 204, 212, 239, 241, and 276. This result is similar to the study performed by Dewi [16], where the sequencing results of the *MIC3* gene on pGEMT (used as the positive control in this study) was analyzed using the BLAST program. Alignment of the pWTA-M3 sequenced using the pUC/M13 forward primer showed 97% homology with the *MIC3* gene of the RH isolate from base 676 up to base 1164. The sequence obtained by pUC/M13 reverse primer showed 98% homology from 1387 bp to 1833 bp.

Martin *et al*. [10] stated that the DNA vaccine vector which expressed the GRA-4 whole protein has been designed and used in immunization tests to compare the protective value of a vaccine by rGRA-4 which has been combined with alum. The authors also suggested that GRA-4 is a good candidate in vaccine development against toxoplasmosis.

Electrophoresis of GRA-4 recombinant protein using SDS PAGE (Figure-5) showed the protein recombinant with molecular weight of 48 kDa. This data differed from data observed by Ram *et al*. [23] and Meng *et al*. [24]. The difference in molecular weight is related to the histidine-tagged application [18]. The HEK293T eukaryotic cell was used for gene expression and found that the molecular weight was about 70 kDa, which was composed of 40 kDa and 30 kDa weights of *GRA-4* gene expression and green fluorescent protein producer, respectively [24].

This study presented the preliminary data regarding the production of recombinant antigen GRA-4 of *T. gondii*, which showed the ability to be cloned into the pET SUMO plasmid. The sensitivity of GRA-4 cloning in pET SUMO plasmid will be tested in mice in future studies. The final target of our study is to develop the diagnostic kit for toxoplasmosis using the cloned *GRA-4* in pET SUMO plasmid.

## Conclusion

Here, we cloned the *GRA-4* gene from a locally obtained *T. gondii* isolate into the pET SUMO plasmid. The amplification resulted in a 1036 bp *GRA-4* gene fragment, which produced a 48 kDa molecular weight after expression in DE3 competent cells.

## Authors’ Contributions

MH supervised the overall research work. TZH, AS, DP, and FF participated in sampling, made available relevant literatures, executed the experiment, and analyzed the cloning and expression of *T. gondii* GRA-4 recombinant protein. All authors interpreted the data, critically revised the manuscript for important intellectual contents, and approved the content.
